# Characterization of the prohormone complement in *Amphiprion* and related fish species integrating genome and transcriptome assemblies

**DOI:** 10.1371/journal.pone.0228562

**Published:** 2020-03-12

**Authors:** Bruce R. Southey, Sandra L. Rodriguez-Zas, Justin S. Rhodes, Jonathan V. Sweedler

**Affiliations:** 1 Department of Animal Sciences, University of Illinois at Urbana-Champaign, Urbana, Illinois, United States of America; 2 Neuroscience Program, University of Illinois at Urbana-Champaign, Urbana, Illinois, United States of America; 3 Department of Statistics, University of Illinois at Urbana-Champaign, Urbana, Illinois, United States of America; 4 Department of Psychology, University of Illinois at Urbana−Champaign, Urbana, Illinois, United States of America; 5 Department of Chemistry, University of Illinois at Urbana−Champaign, Urbana, Illinois, United States of America; Universite de Rouen, FRANCE

## Abstract

The *Amphiprion* (anemonefish or clownfish) family of teleost fish, which is not a common model species, exhibits multiple unique characteristics, including social control of body size and protandrous sex change. The social changes in sex and body size are modulated by neuropeptide signaling pathways. These neuropeptides are formed from complex processing from larger prohormone proteins; understanding the neuropeptide complement requires information on complete prohormones sequences. Genome and transcriptome information within and across 22 teleost fish species, including 11 *Amphiprion* species, were assembled and integrated to achieve the first comprehensive survey of their prohormone genes. This information enabled the identification of 175 prohormone isoforms from 159 prohormone proteins across all species. This included identification of 9 CART prepropeptide genes and the loss of insulin-like 5B and tachykinin precursor 1B genes in *Pomacentridae* species. Transcriptome assemblies generally detected most prohormone genes but provided fewer prohormone genes than genome assemblies due to the lack of expression of prohormone genes or specific isoforms and tissue sampled. Comparisons between duplicate genes indicated that subfunctionalization, degradation, and neofunctionalization may be occurring between all copies. Characterization of the prohormone complement lays the foundation for future peptidomic investigation of the molecular basis of social physiology and behavior in the teleost fish.

## Introduction

Non-model species provide a unique opportunity to gain a more complete understanding of the molecular mechanisms underlying processes such as sex change that are not observed in classical model species [1]. The increasingly affordable and accurate genome and transcriptome next-generation sequencing technologies are benefiting the study of these non-model organisms [1]. Members of the *Amphiprion* (anemonefish or clownfish) genus are examples of a non-model model species for which the research interest and opportunity for affordable next-generation sequencing has ensured the availability of several genome and transcriptome datasets for these teleost fish species.

*Amphiprion* are mainly found in tropical Indo-West Pacific regions and live in a symbiotic relationship with certain sea anemone species [2]. A unique characteristic of the 30 *Amphiprion* species is their socially controlled sex and corresponding body size changes. They typically live as a small social group in a size-based socially controlled dominance hierarchy where the largest individual is the adult female and the second largest is the adult male, with varying numbers of immature individuals of differing sizes, depending on species and anemone host size [2, 3]. When the female is lost, the male changes to female, typically over a period of one to three months [2, 4–7]. In some species, the largest non-adult individual can move to a new host to become a female [2–8]. When the male either undergoes the protandrous sex change or is lost, the largest non-adult replaces the male. The body size ratio between individuals is socially maintained as a constant function of the host anemone size [9]. This unique feature of the *Amphiprion* species may have evolved as a response to limited shelter space and reproductive competition [10].

Underlying the *Amphiprion* socially driven sex and body size changes is a complex array of biological signals and changes that involves reorganization of the chemistry and structure of the brain, genitalia, gonads, body size, and body features [11, 12]. These biological signals are modulated by neuropeptides, small signaling molecules present in the brain and hypothalamic-pituitary-gonadal axis. Examples of these signaling molecules include the pituitary prohormone genes, notably neuropeptide VF precursor (NPVF or gonadotropin-inhibitory hormone GnIH), proopiomelanocortin (POMC), Oxytocin (OXT) and Vasopressin (AVP) [3, 11, 13]. In fish NPVF exerts numerous reproductive inhibitory effects as well as effects on behavior [14]. The melanocortin system involving the POMC genes have been shown to be directly related to cichlid behavior where yellow *Astatotilapia burtoni* males were more aggressive and had lower cortisol levels than the blue males [15]. Both AVP and OXT genes contain similar nonapeptides, isotocin (homologous to mammalian oxytocin) and vasotocin (homologous to mammalian vasopressin), respectively, that influence various fish social behaviors [8, 11, 16–21]. AVP has been associated with a variety of fish behaviors, including social status [22, 23], and regulates mate-guarding behaviors [24] and species differences in mutualistic behavior [25]. In contrast, OXT has been less studied but appears to be a regulator of social response [23], such as male courtship [26], mediating social habituation [27], and may be associated with sexual dimorphism [25].

Neuropeptides are formed by enzymatic cleavage and chemical post-translational modifications of larger, inactive precursor proteins referred to as prohormones. In order to characterize these neuropeptides, the prohormone complement first needs to be revealed using sequence information and a list of known prohormones [28–34]. Ideally, the sequence information is obtained from the genome sequencing project of the species of interest but next-generation sequencing of the transcriptome can also be used for identification [35]. When there is no sequence information for the species of interest, genome and transcriptome sequence information can be obtained from closely related species. Integration of next-generation sequencing of genome and transcriptome sequence information across species would then provide accurate and comprehensive gene annotation and characterization.

The investigation of neuropeptides that control socially-driven physiological changes has been hindered by the limited genome and transcriptome sequence resources available for *Amphiprion* and related species. The first objective of the present study was to develop a comprehensive survey of the prohormone complement in *Amphiprion ocellaris*. The detailed and comprehensive sequence information secured upon the accomplishment of our first objective enabled us to pursue a second objective, the characterization of the prohormone complement of the more distantly related *Pomacentridae* (Damselfish) family that includes the *Amphiprion* genus within the *Perciformes* (Perch-like) order. The final objective was to compare the prohormone complement across species to identify unique and common sequences that could offer insights into unique and common physiological and behavioral features. A supporting goal was the development of a bioinformatics pipeline that integrates genome and transcriptome sequence information across species. An innovative bioinformatics workflow was developed to address the fragmentary nature of the sequencing information available and to integrate complementary genomic and transcriptomic sequence records across *Amphiprion* species.

The exhaustive survey of prohormone sequences across fish families obtained from the present inter-species and multi-step bioinformatics initiative enable the accurate identification and quantification of corresponding genes, proteins and peptides. This information supports the study of molecular mechanisms regulated by neuropeptides, such as behavior, body size, sex plasticity and feeding [8, 18–21, 36–38]. Moreover, the multi-species comparison enabled the identification of conserved regions among the prohormones, and sequences that are unique to particular species or families. The results from the present comparative omics study may aid further work to understand both function and evolutionary changes associated with neuropeptide specialization and species divergence.

## Material and methods

### Bioinformatic annotation of prohormones across species

**Fig 1:**
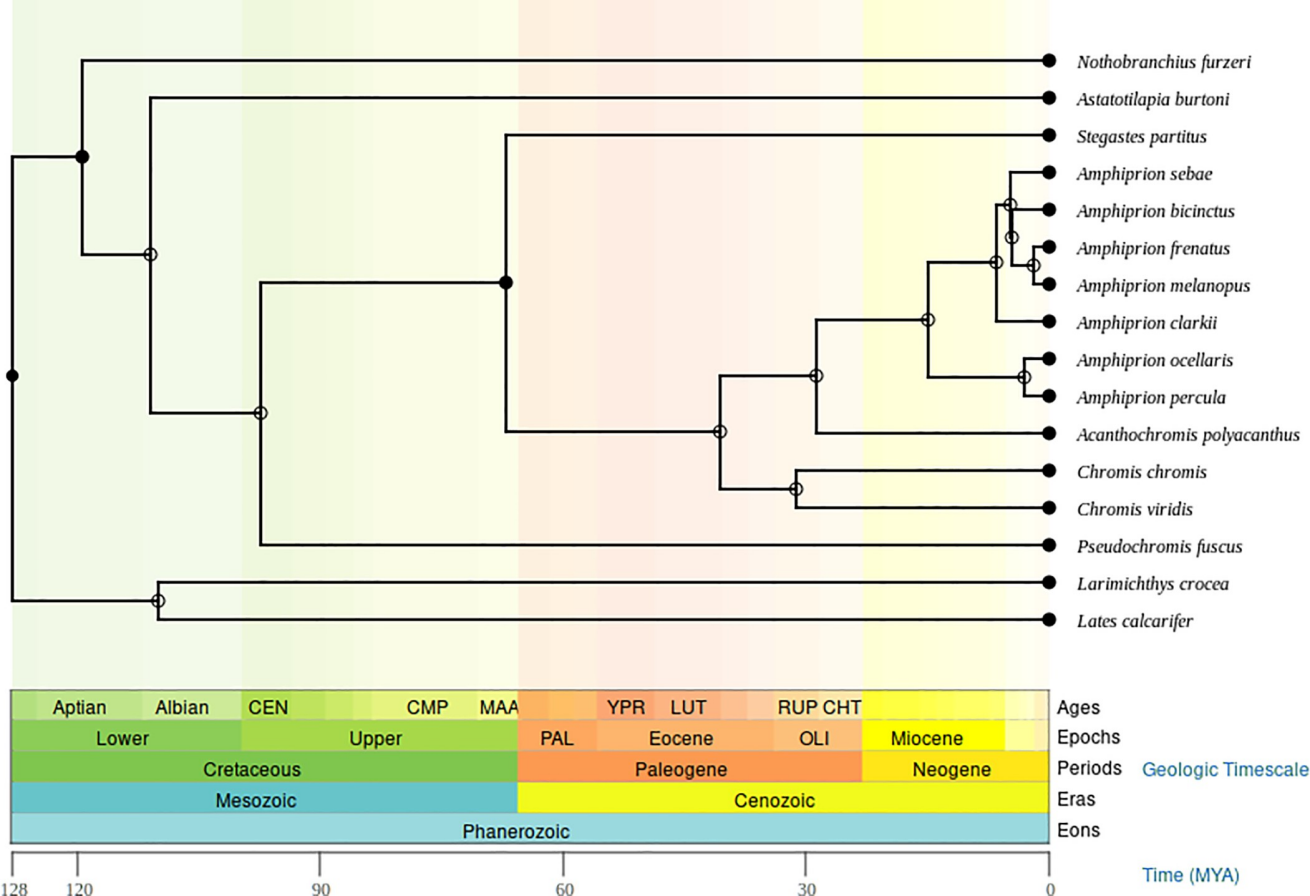
Evolutionary relationship of 16 *Percomorphaceae* species studied.

Genome and transcriptome sequence information from 22 species, including 11 *Amphiprion* species, were obtained from published sources or publicly available databases, including National Center for Biotechnology Information (NCBI) Biosystems databases [39]. Information was obtained from the following specific species: *Amphiprion* species with sequenced DNA genomes by subgenus *Actinicola*: *Amphiprion ocellaris* (3 assemblies) [40–42], *Amphiprion percula* [40, 41]*; Amphiprion clarkii-complex*: *Amphiprion bicinctus* and *Amphiprion clarkia* [42]*; Amphiprion ephippium-complex*: *Amphiprion frenatus* [43] and *Amphiprion melanopus* [42]; *Paramphiprion*: *Amphiprion polymnus* [42] and *Amphiprion sebae* [42]; *Phalerebus*: *Amphiprion akallopisos*, *Amphiprion nigripes* and *Amphiprion perideraion* [42]; and *Amphiprion* species with transcriptome data: *A*. *bicinctus* [44], *A*. *clarkia* [41], *A*. *melanopus* [45], *A*. *percula* [40, 41], *A*. *ocellaris* and *A*. *sebae*. Species from the *Pomacentridae* family with DNA genome data were *Acanthochromis polyacanthus*, *Chromis chromis*, *Pomacentrus moluccensis* [42], *Premnas biaculeatus* [42] and *Stegastes partitus*. Transcriptome data were from *Chromis viridis* [41]. *Ovalentaria* was represented by the DNA genome assemblies of *Astatotilapia burtoni* [46] and *Pseudochromis fuscus* [47]. Three species from the Perciform order (*Percomorphaceae*) with sequenced DNA genome assemblies selected based on similarity were: protandrous hermaphrodite *Lates calcarifer* [6, 48], *Larimichthys crocea* [49], and *Nothobranchius furzeri* [50]. The estimated divergence time from *Amphiprion* genus from other genera in the *Pomacentridae* family is between 12 and 36 mya, from the *Ovalentaria* group was 95 mya, and from Perciform between 95 to 126.8 mya [51, 52]. The phylogenetic tree with evolution time between species studied (Fig 1) was obtained from TimeTree [52].

The genome and transcriptome sequences of all the species studied were obtained from the appropriate NCBI Bioproject (Table 1). For species without available assemblies, *de novo* assemblies were obtained using MEGAHIT [53], SOAPdenovo [54], and Trinity [55, 56] with default settings and without any preprocessing of reads for quality.

**Table 1:** Number and protein sequence similarity of prohormone isoforms identified from genome and transcriptome assemblies.

Series	Family	Species	Subgenus	Assembly^a^			Deg. Sim.^b^			
				T	BioProject	Version				
							E	C	P	M
*Ovalentaria*	*Pomacentridae*	*Amphiprion ocellaris*	*Actinicola*	G	PRJNA407816	AmpOce1.0	35	63	72	5
				H	PRJNA515163	42	34	63	72	6
				I	PRJNA515163	42	27	52	87	9
				T	PRJNA374650	de novo	26	53	79	17
		*Amphiprion percula*	*Actinicola*	G	PRJNA436093	Nemo_v1.1	35	65	70	5
				T	PRJEB27750	de novo	33	53	78	11
		*Amphiprion bicinctus*	*Amphiprion clarii-complex*	G	PRJNA515163	42	34	63	72	6
				T	PRJNA294760	de novo	33	55	61	26
		*Amphiprion clarkii*	*Amphiprion clarii-complex*	T	PRJEB27750	de novo	28	46	70	31
		*Amphiprion frenatus*	*Amphiprion ephippium-complex*	G	PRJNA433458	43	33	63	72	7
		*Amphiprion melanopus*	*Amphiprion ephippium-complex*	G	PRJNA515163	42	34	63	72	6
				T	PRJNA398732	de novo	7	9	53	106
		*Amphiprion polymnus*	*Paramphiprion*	G	PRJNA515163	42	34	63	72	6
		*Amphiprion sebae*		G	PRJNA515163	42	34	62	72	7
				T	PRJNA285007	de novo	30	53	60	32
		*Amphiprion akallopisos*	*Phalerebus*	G	PRJNA515163	42	34	62	71	8
		*Amphiprion nigripes*		G	PRJNA515163	42	34	63	72	6
		*Amphiprion perideraion*		G	PRJNA515163	42	35	62	71	7
		*Pomacentrus moluccensis*		G	PRJNA515163	42	35	62	71	7
		*Premnas biaculeatus*		G	PRJNA515163	42	34	60	73	8
		*Acanthochromis polyacanthus*		G	PRJNA311159	ASM210954v1	35	61	73	6
		*Chromis chromis*		G	PRJEB12469	de novo	33	64	69	9
		*Chromis viridis*		T	PRJEB27750	de novo	27	41	75	32
		*Stegastes partitus*		G	PRJNA89147	Stegastes_partitus-1.0.2	38	65	67	5
*Ovalentaria*	*Pseudochromidae*	*Pseudochromis fuscus*		G	PRJEB12469	47	26	48	91	10
*Ovalentaria*	*Cichlidae*	*Astatotilapia burtoni*		G	PRJNA60363	AstBur1.0	32	65	72	6
*Ovalentaria*	*Nothobranchiidae*	*Nothobranchius furzeri*		G	PRJEB5837	Nfu_20140520	24	66	63	22
*Carangaria*	*Centropomidae*	*Lates calcarifer*		G	PRJNA345597	ASM164080v1	36	75	57	7
*Eupercariai*	*Sciaenidae*	*Larimichthys crocea*		G	PRJNA245366	ASM74293v1	28	74	59	14

^a^ Assembly where T: Type of sequence data denoting if the sequence is from the genome (G), alternative genome assembly (H), de novo genome assembly (I) or transcriptome (T); BioProject: NCBI BioProject identifier; Version: provides the assembly identifier, reference for the assembly or denotes that a de novo assembly was used.

^b^ Deg. Sim: Degree of Similarity of sequences across species where Exact (E)–sequences aligned without gaps; Close (C)–sequences aligned with less than 5 consecutive gaps; Partial (P)–more than 5 consecutive gaps within the alignment; Missed (M)–No prohormone isoform annotated.

Prohormone annotations were obtained using our published prohormone prediction pipeline [29, 34] using the *A*. *burtoni* annotated prohormone complement [33]. Briefly, a TBLASTN [57] search of each *A*. *burtoni* prohormone sequence was performed on each genome or transcriptome assembly. For genome assemblies, GeneWise [58] was used to predict the protein sequence from region with the best match and the *A*. *burtoni* sequence. For the transcriptome assemblies, the matched RNA sequence was translated into a protein sequence and the open reading frame(s) corresponding to the prohormone sequence were extracted. When a complete sequence was not obtained from a single RNA transcript due to incomplete sequence coverage, GeneWise and homology based on the TBLASTN match were combined to predict the most likely sequence based on the prohormone sequences from *A*. *burtoni* and other species. Prohormone isoforms were identified where different protein sequences were observed in at least one species that were not due to incomplete sequence coverage and present either in at least one transcriptome assembly. These prohormone isoforms were also predicted using GeneWise in the other species. Each predicted prohormone sequence was evaluated for the presence of signal peptide using SignalP [59] and potential cleavage sites using NeuroPred [60]. Prohormone sequences were verified from multiple sequence alignments performed with the L-INS-i iterative refinement method MAFFT v6.861b [61]. Completeness of the *A*. *ocellaris* and *A*. *percula* prohormone isoforms and the *Amphiprion* transcriptome assemblies were assessed by the number of gaps in MAFFT multiple sequence alignments. Within species with multiple assemblies, a single sequence with the fewest gaps was used for subsequent analysis.

Phylogenetic trees were obtained using Phyml 3.1 [62] with default settings from the multiple sequence alignments for all sequences (*Pomacentridae* and *Amphiprion*). The evolution rate of change for each prohormone isoform was computed using Mean Protein Evolutionary Distance (MeaPED) [63], 100 times the mean distance between species divided by the median length of the sequences used. Normalized Robinson–Foulds metric (nRF) was calculated for each prohormone isoform as Robinson–Foulds metric [64] divided by the maximum Robinson–Foulds metric observed. Proportion of edges (Pedges) shared between phylogenetic trees were averaged for each prohormone isoform. Mean distance between species, Robinson-Foulds metric between phylogenetic trees, and proportion of edges between phylogenetic trees were computed using ETE 3 [65].

Multiple sequence alignment was performed with the L-INS-i iterative refinement method MAFFT v6.861b for the CARTPT (sequences provided in S2 File CARTPT sequences) and PCSK1N (sequences provided in S3 File PCSK1N sequences). The phylogenetic tree of CARTPT was obtained from Phyml 3.1 [62] with default settings from the multiple sequence alignment and generated by using ETE 3 [65].

## Results

### Identification of the teleost prohormone complement

Existing or *de novo* genome or transcriptome assemblies were used to annotate the prohormone complement from 22 species, including 11 *Amphiprion* species in the *Percomorphaceae* subdivision of teleost fish (Table 1; Fig 1). Three different genome assemblies and 1 transcriptome assembly were used for *A*. *ocellaris* and 4 other species with both genome and transcriptome assemblies. For the remaining species, genome (15 species) and transcriptome (2 species) assemblies were used. All genome and transcriptome assemblies available from the same species were from different sequencing studies or used different sequencing data.

Across all 22 species, 175 prohormone isoforms from 159 prohormones were identified (Table 1), with individual sequences provided in S1 File. Species with only transcriptome assemblies were limited to the particular prohormone isoforms present and, consequently, many isoforms were not identifiable from some transcriptome assemblies. Among the prohormones with multiple isoforms, 146 prohormones had only one sequence, 12 prohormones were detected with two isoforms, and one prohormone, neuromedin U (NMU), was detected with 5 isoforms.

Evidence of differential gene loss across species was identified for 3 neuropeptide genes: augurin 2 (AUGN2), insulin-like 5B (INSL5B), and tachykinin precursor 1B (TAC1B). Both INSL5B and TAC1B appear to have been lost in the *Pomacentridae* after *Pomacentridae* diverged from the other families from the *Ovalentaria* series. AUGN2 was only detected in the some of *Pomacentridae* species (complete sequences in *S*. *partitus* and *C*. *chromis* and partial sequences in *C*. *viridis*, *A*. *percula* and *A*. *ocellaris*), indicating that it may have been retained in *Pomacentridae* or arose from tandem duplication in *Pomacentridae*.

**Fig 2:**
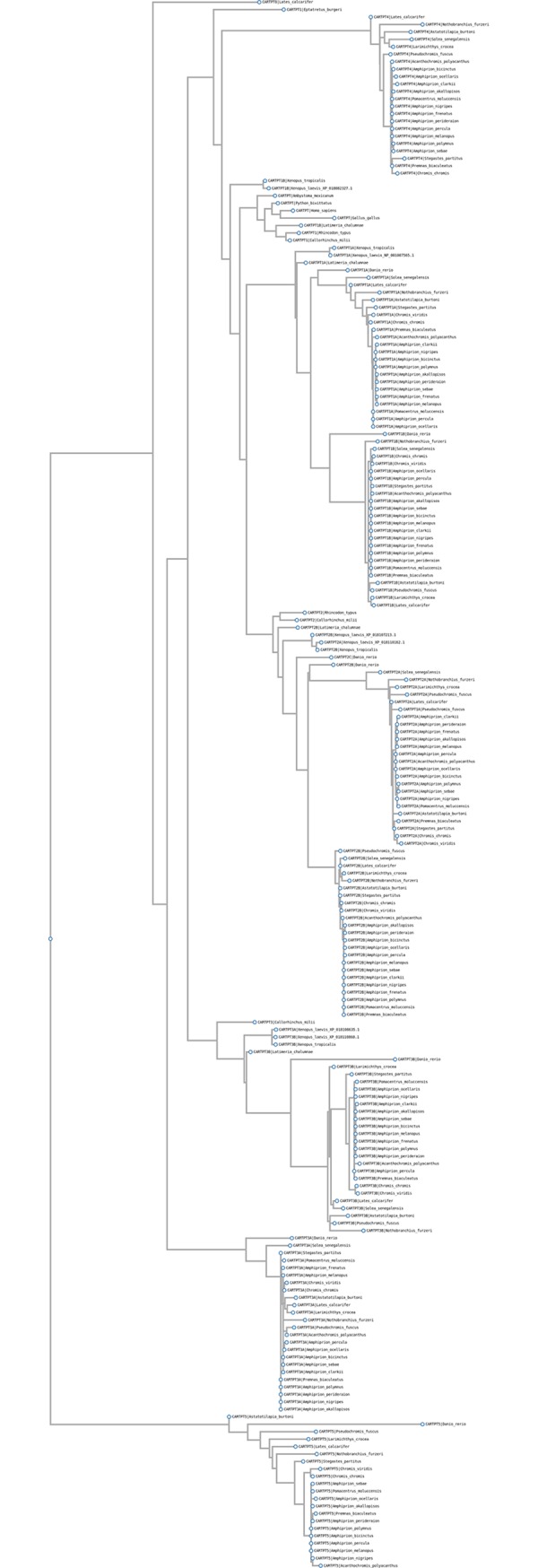
Phylogenetic tree of the fish CART prepropeptide (CARTPT) prohormone protein sequences.

Multiple copies of the CART prepropeptide (CARTPT) and hepcidin antimicrobial peptide (HAMP) gene families were detected. Among the 9 CARTPT genes detected, only CARTPT2A and CARTPT3A were detected in all species (Fig 2). A partial fragment of an additional CARTPT gene (CARTPT8) was only detected in *L*. *calcarifer*. Several hepcidin antimicrobial peptide (HAMP) genes were detected but not all HAMP genes were detected in all assemblies. Only HAMP1 was detected in all assemblies and HAMP3A and HAMP4 were not annotated in any transcriptome assemblies. The *A*. *ocellaris* HAMP3C sequence was only located in the transcriptome assembly and was not detected in any other *Pomacentridae* genome or transcriptome assemblies, implying inconsistencies in the sequencing and assembly.

### Impact of assembly type and species on the detection of prohormones

The evaluation of multiple sequence alignments indicated that the determination of recovery was confounded with taxonomic and assembly related differences. There was inadequate experimental evidence to confirm that general trends were apparent from the detection of proteins across species. Table 1 summarizes the data for all species studied based on the number of consecutive gaps within the alignment of all species, with individual prohormone isoforms given in S1 Table. Genome assemblies generally provided fewer missing prohormone isoforms and higher recovery of complete sequences than the transcriptome assemblies. Excluding prohormones not detected within taxonomic groups, no genome assembly provided all identified prohormone isoforms. Most *Pomacentridae* species lacked HAMP4C, which was only detected in the *A*. *ocellaris* transcriptome assembly.

Assembly quality was a more important factor than the species and source of data used in prohormone detection. The *A*. *melanopus* transcriptome assembly was derived from gill samples and, thus, lacked most of the expected prohormones, and most prohormone predictions were incomplete. The *N*. *furzeri* genome assembly provided a number of unpredicted and incomplete prohormone isoforms similar to many of the transcriptome assemblies. The *A*. *bicinctus* and A. *sebae* transcriptome assemblies supported a similar or greater recovery rate of complete prohormones to the genome assemblies from other species.

The impact of data types on the assembly and subsequent prohormone gene detection was assessed from the comparison of the genome and transcriptome assemblies of *Amphiprion* species (Table 2). Overall, 60% of the prohormone isoforms were completely recovered from all transcriptome assemblies with either the same sequence or amino acid variants. Only 14% of the prohormone isoforms detected in the genome were missed by the corresponding transcriptome assembly. Some prohormone isoforms missed in the corresponding genome assemblies were detected in the transcriptome assemblies. However, 14% of the prohormone isoform transcriptome predictions had alignment gaps compared to 6% of prohormone isoform genome predictions.

**Table 2:** Comparison between different assemblies from the same species.

Species 1		Species 2		Sequence^a^		Gap^b^			Missing^c^		
Name	At^d^	Name	At	Identity	Variant	Both	Sp1	Sp2	Both	Sp1	Sp2
*Amphiprion ocellaris*	G	*Amphiprion ocellaris*	H	50	81	4	7	27	5	0	1
*Amphiprion ocellaris*	G	*Amphiprion ocellaris*	I	54	66	6	3	37	5	0	4
*Amphiprion ocellaris*	H	*Amphiprion ocellaris*	I	79	27	14	16	30	6	0	3
*Amphiprion bicinctus*	G	*Amphiprion bicinctus*	T	80	38	3	14	14	6	0	20
*Amphiprion melanopus*	G	*Amphiprion melanopus*	T	8	11	3	2	45	6	0	100
*Amphiprion ocellaris*	G	*Amphiprion ocellaris*	T	79	32	3	5	38	4	1	13
*Amphiprion ocellaris*	H	*Amphiprion ocellaris*	T	37	54	6	22	37	4	2	13
*Amphiprion ocellaris*	I	*Amphiprion ocellaris*	T	42	44	13	25	29	4	5	13
*Amphiprion percula*	G	*Amphiprion percula*	T	61	66	2	3	32	5	0	6
*Amphiprion sebae*	G	*Amphiprion sebae*	T	76	31	4	11	20	6	1	26

^a^ Sequence: Sequences aligned without gaps with either complete sequence identity or presence of amino acid variants.

^b^ Gap: Sequence aligned with at least one gap in both sequences (Both), only in the first species (Sp1) or only in the second species.

^c^ Missing: No sequence recovered from corresponding assembly from both species (Both), only in the first species (Sp1) or only in the second species.

^d^ Assembly molecular type: G–genome assembly; H–[42] alternative genome assembly; I–[42] de novo genome assembly; T–de novo transcriptome assembly.

Comparisons across species and assemblies identified probable assembly errors (e.g., unanticipated stop codons) and gaps (e.g., variation in the number of sequentially repeated amino acids). Direct comparison of predictions from the *A*. *percula* and *A*. *ocellaris* assemblies identified a small region of secretogranin IIB (SCG2B) that differed between genome and transcriptome. The partial aligned sequence, TEES**D-**AKAAQGI, from the *A*. *percula* and 2 of the *A*. *ocellaris* genome assemblies was one amino acid shorter from the corresponding partial aligned sequence, TEES**ES**AKAAQGI, from the *A*. *ocellaris* de novo assembly and the *A*. *percula* and *A*. *ocellaris* transcriptome assemblies. The corresponding region was present in the actual raw data and was identified as a 3 nucleotide insertion or deletion (indel) in the respective assemblies.

The recovery of prohormone isoform sequences from the *Amphiprion* transcriptome assemblies varied between species due to gene expression and sequence coverage (Table 3). Overall 73% of all prohormone isoforms (128 isoforms) were completely recovered from at least 2 *Amphiprion* transcriptome assemblies. However, the recovery of any specific prohormone isoform varied across species with 14 prohormone isoforms detected in all 6 *Amphiprion* species. Allowing for 1 missed species, 68% of all prohormones were completely recovered in at least 3 species and partially recovered in the remaining species. In addition to the 4 prohormone isoforms not found in *Amphiprion* species, HAMP3B and NMU isoform 6 (found in the *C*. *viridis* transcriptome assembly) were not detected in any transcriptome assembly but were detected in the 3 *Amphiprion* species that had genome assemblies.

**Table 3:** Number of prohormone isoforms recovered from the different *Amphiprion* de novo transcriptome assemblies.

Complete^a^	Partial^b^	Missing^*c*^			Total
		0	1–2	3+	
3+	0	14	23	3	40
3+	1	27	21	1	49
3+	3+	10	11	0	21
2	0	0	0	2	2
2	3+	5	4	7	16
1	0	0	0	6	6
1	1	0	0	5	5
1	2	0	0	5	5
1	3+	5	5	6	16
0	0	0	0	6	6
0	3+	4	2	3	9
Total		65	66	44	175

^a^ Number of prohormone isoforms that were completely recovered from 0, 1, 2 or 3 or more species.

^b^ Number of prohormone isoforms that were partially recovered from 0, 1, 2, or 3 or more species.

^c^ Number of prohormone isoforms that were not recovered from 0 species, 1 or 2 species, or 3 or more species.

### Changes in prohormone evolution distance

The average nRF and Pedges in common between trees in prohormone families with at least 5 protein isoforms indicated that 62 to 73% of the phylogenetic trees had similar topology regardless of prohormone family (Table 4). Comparison of individual protein isoforms (S2 Table) from these prohormone families exhibited variation between individual members often related to differences between duplicated genes. The average MeaPED varied across prohormone families with at least 5 protein isoforms, indicating that prohormone families had different levels of conservation (Table 4). Nucleobindin, Granin and PDGF/VEGF growth factor families all exhibited high conservation across multiple genes. However, the Parathyroid, Tachykinin and Hepcidin families exhibited large variation between members. Within the Hepcidin family, HAMP2, HAMP3A, HAMP3B, HAMP3C and HAMP4A had greater MeaPED than HAMP1. INSL3 exhibited high MeaPED in *Pomacentridae* and *Amphiprion* species but average change when considered with all species. While TAC1B was not detected in the *Pomacentridae* species, a relatively high rate of MeaPED was observed that indicated this gene was altered between species.

**Table 4:** Average Mean Protein Evolutionary Distance (MeaPED), normalized Robinson–Foulds metric (nRF) and proportion of edges shared between phylogenetic trees (Pedges) for prohormone families with 6 or more prohormones for *Amphiprion*, *Pomacentridae* and All species.

Family	N isoforms	MeaPED			nRF			Pedges		
		*Amphiprion*	*Pomacentridae*	All	*Amphiprion*	*Pomacentridae*	All	*Amphiprion*	*Pomacentridae*	All
Calcitonin	10	0.012	0.032	0.106	0.88	0.84	0.83	0.61	0.61	0.61
CARTPT	9	0.011	0.036	0.116	0.91	0.88	0.86	0.60	0.59	0.59
Corticotrophin	5	0.007	0.036	0.085	0.88	0.87	0.83	0.61	0.59	0.61
Endothelin sarafotoxin	6	0.011	0.053	0.173	0.89	0.81	0.80	0.60	0.62	0.62
Glucagon	10	0.024	0.042	0.107	0.88	0.91	0.88	0.61	0.58	0.58
Granin	9	0.004	0.012	0.037	0.82	0.71	0.71	0.64	0.67	0.67
Hepcidin	7	0.047	0.189	0.605	0.85	0.90	0.87	0.63	0.59	0.60
Insulin	6	0.008	0.034	0.119	0.84	0.85	0.81	0.63	0.61	0.62
Natriuretic peptide	6	0.007	0.044	0.141	0.87	0.83	0.78	0.61	0.62	0.63
Neuromedin U (NmU)	6	0.011	0.036	0.124	0.85	0.83	0.76	0.63	0.62	0.64
Opioid	8	0.006	0.024	0.071	0.82	0.78	0.74	0.64	0.64	0.65
Parathyroid	7	0.015	0.055	0.165	0.88	0.82	0.79	0.61	0.62	0.63
PDGF/VEGF growth factor	16	0.004	0.016	0.054	0.88	0.83	0.78	0.61	0.62	0.63
Relaxin	6	0.031	0.071	0.253	0.85	0.86	0.74	0.63	0.60	0.66
Somatostatin/Urotensin	9	0.013	0.046	0.142	0.87	0.88	0.82	0.61	0.59	0.61
Tachykinin	7	0.018	0.061	0.250	0.84	0.83	0.76	0.63	0.61	0.66
Total	175	0.015	0.046	0.143	0.87	0.84	0.80	0.62	0.61	0.62

## Discussion

### Prohormone complement

Genome availability of *Amphiprion* and related species enabled the annotation of prohormones across species using different sequencing technologies. Compared to the *A*. *burtoni* prohormone complement [33], INSL5B and TAC1B were lost in the *Pomacentridae* species and AUGN2 was lost in the non-*Pomacentridae* species; multiple CARTPT and HAMP genes and the additional somatostatin 6 (SST6) gene, also identified in *Danio rerio* [66], were detected. Differences in the number of HAMP genes detected between species are likely due to genome-wide and species-specific tandem duplication and, as evident with *A*. *ocellaris* HAMP3C, sequence assembly and prediction.

The detected differences in the prohormone complement between *Amphiprion* and *Pomacentridae* species could be associated with differences in the physiology of these fish groups that have potential impact in their social behavior. For example, sound production could be a component of courtship and agonistic behaviors in fish species, and significant sexual dimorphism in physiology and morphology have been linked to sonic/vocal pathways. Only males produce sound in many species of pomacentrids and cichlids, whereas females can produce similar sounds in cichlid species [67]. The loss of TAC1B detected in our study could be associated with the differential social behavior between *A*. *burtoni* and the *Pomacentridae* species and with reports of the effect of injections of a tachykinin receptor antagonist in *Carassius auratus* [16, 68]. Peripheral injections of a tachykinin receptor antagonist completely blocked the effects of central arginine vasotocin on the social approach behavior of *C*. *auratus* whereas central infusions of the tachyninin receptor antagonist had no effect on social approach [16, 68].

The absence of INSL5B in the *Pomacentridae* species and presence in the *A*. *burtoni* genome could explain the differences in behavior between these species. INSL5B shares receptors with Relaxin 3 (RLN3), a prohormone that presents broadly similar expression patterns in *Oryzias latipes* and *D*. *rerio* [69]. *Oryzias latipes* is highly sensitive to environmental stimuli and studies have postulated the role of INSL5B and RLN3 in modulating the effect of stress on reproduction, growth and feeding behavior [69]. The impact of the loss of AUGN2 in the non-*Pomacentridae* species on social behavior cannot be completely assessed due to the limited study of this prohormone in fish. In *D*. *rerio*, AUGN2 but not AUGN1 was associated with hypoxia response in embryos and embryonic brain hypoxia has been related to behavioral changes [70].

CARTPT produces neuropeptides that are involved with multiple biological processes, including stress, feeding, and reward dependencies [36, 71]. The present study increased the number of reported CARTPT teleost fish genes [33, 72–74] to 9. The CARTPT fish gene sequences (Fig 2) are consistent with two rounds of vertebrate whole genome duplication, the teleost whole genome duplication and tandem duplication [74]. Within *Cyclostomata* (jawless vertebrates) species, a partial CARTPT sequence was detected in the *Eptatretus burgeri* genome assembly. Within the *Chondrichthyes* (cartilaginous fishes), 2 and 3 CARTPT sequences were found in the draft *Rhincodon typus* and *Callorhinchus milii* assemblies, respectively. Further duplication of the CARTPT genes occurred with *Euteleostomi* (bony vertebrates) since duplicated genes are found in both *Actinopterygii* (ray-finned fishes) and *Sarcopterygii* superclasses. The role of these multiple CARTPT genes is unclear because the majority of studies in fish have not examined the 8 completely annotated CARTPT genes. Different CART genes exhibit different expression patterns [73, 75, 76], and differential response to feeding [73, 75], and stress [77].

The differences in number of CARTPT genes between fish species could contribute to differences in social behavior. Territorial intrusion and the associated interactions with neighbors or intruders was correlated with under-expression of the CARTPT gene in the diencephalon of male *Gasterosteus aculeatus* [78, 79]. Likewise, differences in the number of HAMP genes between species could be linked to behavioral differences. The expression of HAMP genes in the mudskipper *Boleophthalmus pectinirostris* has been associated to the behavior of this burrow-dwelling species, characterized by augmentation of the burrow tunnel during the spawning season where a male and female fish mate and lay eggs that are incubated [80]. The HAMP produced in the male reproductive tract have short antimicrobial function and have been postulated to protect the eggs generated from the burrow-bound mating behavior. Lastly, the variation in somatostatin gene number between species observed in the present study has a direct association with variation in social behavior based on the established relationship between this gene family and aggressive, dominant and courtship behavior. Somatostain modulates aggressive behavior and social dominance *A*. *burtoni* [81]. The negative relationship between the neuromodulator somatostatin and aggressive behavior was characterized by increased aggressive behavior with higher levels of somatostatin antagonist whereas somatostatin agonist decreased aggression in *A*. *burtoni*.

The comparison of fish genome and transcriptome sequences enabled the identification of 3 POMC prohormone sequences. Peptides cleaved from POMC prohormones modulate social status [82], stress, color, feeding patterns [83–86], and behavior [87] in fish. A sustained elevation of the expression of the POMC gene observed in subordinates relative to dominant sexually mature rainbow trouts [82]. Also, POMC was over-expressed in *A*. *burtoni* and *Salmo salar* and this pattern was associated with the aggressive and sexual behaviors observed in dominant males relative to subordinate males in these species [88]. The 3 POMC prohormone sequences detected in this study resulted from the teleost whole genome duplication and tandem duplication [89–92]. The tandem duplication of POMC1 appeared to have occurred after *Neoteleostei* split from *Euteleosteomorpha* since only a single POMC1 gene was found in *Protacanthopterygii* genome-sequenced species such as *Esox lucius* (*Esociformes*) or *Oncorhynchus kisutch*, *Oncorhynchus mykiss*, and *S*. *salar* (*Salmoniformes*). The 3 POMC prohormones are precursors of similar corticotropin, melanotropin alpha, and melanotropin beta peptides but different beta-endorphin peptides. The expression of the 3 POMC genes was detected in *A*. *burtoni* [33], *Paralichthys olivaceus* [93], and *Verasper moseri* [94], and peptides were identified in *A*. *burtoni* [33] and *V*. *moseri* [94].

The difference in beta-endorphin sequences between the fish species compared in the present study may be associated with behavioral differences. Low levels of beta-endorphin administration have been associated with higher cohesiveness and duration of schooling and lower latency of school formation whereas higher levels decreased schooling behaviors in *C*. *auratus* [95]. Also, variations in the beta-endorphin sequence across species could influence the effectiveness of receptors to recognize the signal of these endorphins and this could have an effect similar to that of changes in the peptide level.

The prohormone SCG2 has a wide range of functions in fish [96, 97] including neuropeptide release [98] and has been proposed as a signal integrator of glutamate and dopamine inputs [99]. Secretoneurin, EM66, and manserin are mammalian SCG2 peptides that have equivalent peptides in both SCG2A and SCG2B. Secretoneurin is the only SCG2 peptide with known biological effects including reproduction, osmoregulation, hypertension, and stress, and is hypothesized to be co-released with oxytocin and vasopressin [97]. In the electric fish, *Brachyhypopomus gauderio*, the SCG2B secretoneurin was demonstrated to influence electrical behavior [100]. Also, administration of secretoneurin increased feeding behavior and locomotion in the *C*. *auratus* [101].

The secretoneurin peptide is relatively highly conserved between duplicated SCG2 genes detected in some fish species and with the single copy of SCG2 detected in other species [102]. The SCG2B secretoneurin peptide is the only exhibited widespread immunoreactivity in the *C*. *auratus* brain [102, 103]. The differences in the secretoneurin sequence between fish species detected in the present study could be associated with differences in reproductive behavior in consideration of the role of this peptide on the reproductive activity of the catfish *Heteropneustes fossilis* [104]. The observed difference in the SCG2B sequence across fish species identified in this study is located in a peptide resulting from the C-terminal cleavage of the teleost prohormone that is equivalent of the mammalian EM66 peptide. While there is no known matching mammalian peptide, this peptide shows high homology to a predicted mammalian peptide C-terminal to manserin. Multiple sequence alignment showed that the shorter form (TEES**D-**AKAAQGI) of SCG2B was identical to the sequence found in the other *Amphiprion* subgenera. While predictions were supported by the underlying raw data used for the genome and transcriptome assemblies, only the mate pair data from [42] had raw reads from both variants, identifying a 3 nucleotide indel that was possibly due to a sequencing error [105] or natural hybridization in *Amphiprion* [106].

Prohormone convertases process precursor proteins into biologically active peptide. Therefore, the differences in prohormone convertase inhibitor PCSK1N sequences across fish species detected in the present study could be associated to differences in behavior or physiology through differences in the capacity of this molecule to generate biocactive peptides that in turn modulate these characteristics [107]. In mammals, PCSK1N participates in the processing of neuropeptides and also acts as a neural chaperon. Consistent with prior studies [30, 31, 33, 107], PCSK1N prohormone was generally not detected in homology searches within *Sauropsida* and non-Eutherian mammals with genome assemblies. However, a partial *Terrapene mexicana triunguis* PCSK1N prohormone was identified, indicating that PCSK1N may have been lost when *Archosauria* diverged from *Testudines*.

**Fig 3:**
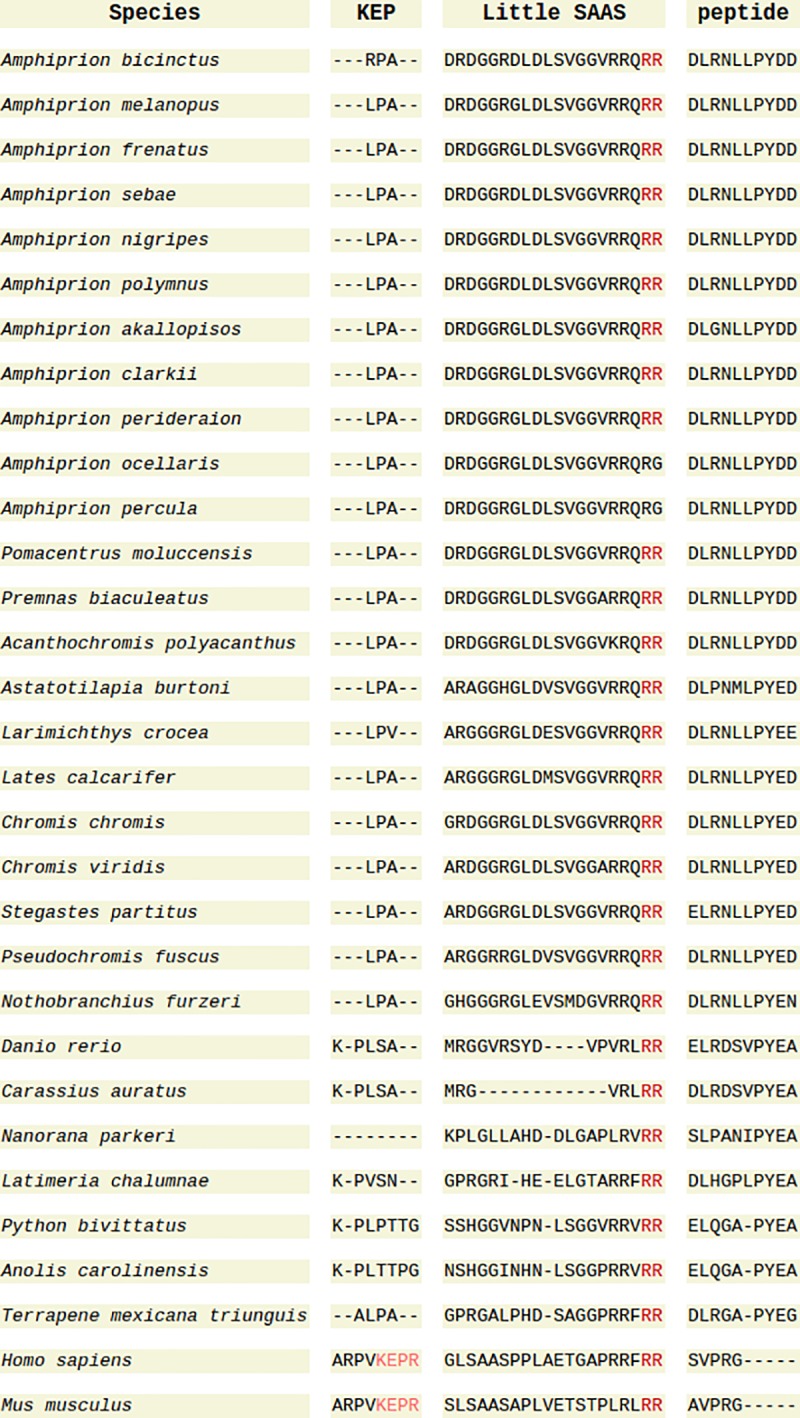
Alignment of Proprotein Convertase Subtilisin/Kexin Type 1 Inhibitor (PCSK1N) prohormone protein sequences containing the mouse KEP and Little SAAS peptides with mouse cleavage sites highlighted.

**Fig 4:**
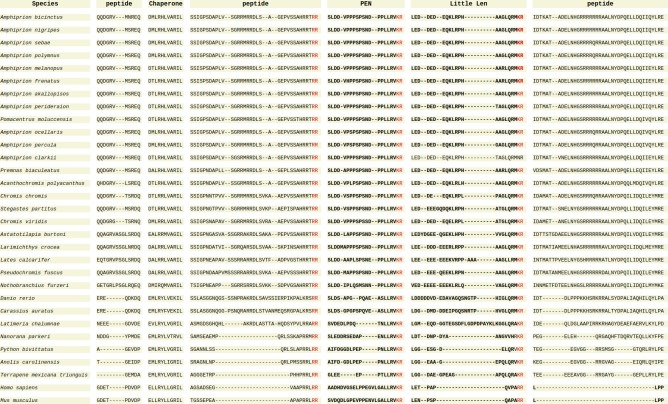
Alignment of Proprotein Convertase Subtilisin/Kexin Type 1 Inhibitor (PCSK1N) prohormone protein sequences containing the conserved chaperone region, the mouse PEN and Little Len peptides, and mouse cleavage sites highlighted.

The alignment of sequences without a signal peptide (Fig 3) shows the locations of mouse-detected peptides KEP and Little SAAS, which are cleaved from the Big SAAS peptide, and the conserved cleavage sites. Both *A*. *ocellaris* and *A*. *percula* lack the cleavage site that produces the Big SAAS peptide and there is no predicted site in the non-mammalian sequences that cleaves Big SAAS into KEP and Little SAAS. The mouse peptide, ELLRYLLGRIL, that is proposed to be essential for the chaperone functionality [108], showed homology in the fish sequences (Fig 4), implying that the peptide may have the same function in teleost fish. The highly conserved convertase inhibitory segment homology region [107] contains the conserved cleavage site that separates the mouse PEN and LEN peptides. The C-terminal cleavage site for PEN is also highly conserved (Fig 4) and peptides similar to the LEN peptide were also reported in *D*. *rerio* [109].

The differences in the number of prohormone gene or protein sequences or variations in the sequences across species identified in the present study could be associated with the differences in physiology and behavior between the fish species studied. Single nucleotide polymorphisms in prohormone genes are known to influence food intake in animal models [110] and could also explain some of the observed differences in physiology and behavior. In addition, molecular differences between fish species that are associated with physiological and behavioral differences could be elicited by differences in epigenetic, regulatory, and post-translational modifications mechanisms affecting the neuropeptide genes studied. Epigenetic mechanisms could be mediators and effectors of environment-dependent sex transitions in fish including the temperature-induced male-female sex reversal in *Thalassoma bifasciatum* [111]. Sex change processes encompass epigenetic reprogramming and intermediate states that present altered epigenetic machinery. *A*. *burtoni* fish that present higher methylation states tend to ascend among the social ranks whereas lower methylation levels were not associated with rank ascension [112]. Also, epigenetics was postulated as a possible cause for the observed shift across generations towards decreased maternal care behavior of young *A*. *burtoni* [113]. The role of epigenetic changes in DNA methylation in the reprogramming of the *N*. *pulcher* hypothalamus-pituitary-I axis and impact on the prohormones produced by this system is being elucidated [114]https://royalsocietypublishing.org/doi/full/10.1098/rspb.2012.2605.

Transcription factors regulate the transcription level of some genes associated with behavior in fish and could contribute to differences in behavior and physiology between species in addition to gene number, variants and epigenetic effects. The minearalcorticoid receptor acts as a transcription factor and can be more effective at regulating gene expression than glucocorticoids in *A*. *burtoni*. Also, two glucocorticoid receptors found in the African cichlid *Neolamprologus pulcher* have been associated with behavior and stress response [114]https://royalsocietypublishing.org/doi/full/10.1098/rspb.2012.2605. A study of the *D*. *rerio* forebrain identified neurons that express cell-type specific combinations of transcription factors that are required for the expression of the neuropeptide vasotocin-neurophysin gene [115].

Post-translational modifications of the prohormones and cleaved neuropeptides can participate, together with gene variants in sequence and copy number, epigenetics and transcriptional regulation, in the modulation of physiological and behavioral characteristics in fish species. This mode of neuropeptide regulation has been studied in gherlin, a neuropeptide identified in the *C*. *auratus*, *O*. *mykiss* and various cichlid species [116]. An acyl post-translational modification of the C-terminal peptide region from the preprogherlin produces the mature gherlin. Also, fish gherlin present a unique amidation posttranslational modification that is not present in mammals or other non-mammalian vertebrates. The physiological functions of ghrelin in fish include the control of drinking behavior, regulation of pituitary hormone secretion, and regulation of food intake [116].

### Effect of resources on prohormone annotation and peptide identification

The study of multiple *Amphiprion* and related species enabled a comparison of the impact of genome and transcriptome resources on prohormone identification. The transcriptome assemblies provided a relatively higher number of incomplete protein sequences compared to the genome assemblies, which was a consequence of the sequence coverage and assembly quality. The relatively poor performance of the *A*. *melanopus* transcriptome assembly is attributed to the use of non-neural gill tissue. One notable advantage of using transcriptome assemblies was the recovery of the complete sequence of multiple prohormone isoforms and novel sequences, such as *A*. *ocellaris* HAMP4C that was unpredicted from the genome assemblies. While *de novo* assemblies generally provided sufficient identification of prohormone genes, gene expression, sequence coverage, sample tissue, and number of samples are also important to extract the complete protein sequence. As a result, some prohormone isoforms were not detected, and incomplete prohormone sequences were recovered in many transcriptome assemblies.

Quality issues were detected in all genome and transcriptome assemblies studied using multiple sequence alignments. The availability of different assemblies from the same species did identify that many of the stop codons and gaps were due to sequencing errors or lack of coverage. The use of multiple *Amphiprion* species enabled the discrimination between assembly errors and taxonomic differences. A notable assembly error was identified in the transcriptome assembly of the *A*. *melanopus* AVP where the OXT signal peptide region was included. This error is likely a direct result of the remarkable degree of similarity between the AVP and OXT genes, even though these genes have resulted from ancient duplication. This issue was resolved by the identification of the actual AVP signal region in the *A*. *melanopus* transcriptome reads and confirmed from the *A*. *melanopus* genome assembly. Utilizing multiple related species also uncovered probable sequencing errors where the number of sequentially repeated amino acids varied between species that cannot be resolved without alternative assemblies.

Sequence differences were associated with differences between species and the genome and transcriptome resources used. These differences are problematic for peptide identification, even with single amino acid variants [117]. The MeaPED rates provide information that can be used to identify miss-specified sequences and improve peptide identification by characterizing the expected sequence similarity between species. Prohormones that have low MeaPED rates are expected to be relatively highly conserved between species such that peptides can be detected using typical across-species comparisons. In contrast, prohormones with low MeaPED rates are expected to be variable and show little sequence conservation. Higher MeaPED rates are consistent with a relatively high rate of evolution, such as INSL3, compared to other members of the teleost relaxin family [118]. These prohormones with relatively high MeaPED rates will require alternative approaches, such as utilizing across species sequence variation [119, 120], to identify proteins and peptides.

Most of 40 prohormone families with retained duplicated genes exhibit similar MeaPED rates between copies, reflecting subfunctionalization where both copies retain the original functionality [121]. In some prohormone families, such as CRH1, NMB, PRLH, spexin hormone (SPX) and TAC3, one copy has over 5 times the rate of the other copy, indicating possible degradation or neofunctionalization [121]. Of the prohormone families that have more than 2 copies, all versions of CARTPT, NUCB and POMC had similar low MeaPED rates, indicating subfunctionalization between copies. Subfunctionalization of POMC is evident by the tissue-specific gene expression profiles of the different *A*. *burtoni* POMC genes [33, 90]. For some prohormones families, such as GCG and INS, subfunctionalization, degradation, and neofunctionalization may be occurring between copies.

## Conclusions

Next-generation sequencing genome and transcriptome sequence information across species was successfully integrated to obtain accurate and comprehensive characterization of the prohormone complement of non-model *Amphiprion* and related species. Direct comparison of species with both genome and transcriptome assemblies showed that the recovery of prohormone sequences was dependent on the sequencing coverage and sample type rather than type of data used. This indicated transcriptome assemblies can be used to provide accurate annotations as well as identify prohormone isoforms resulting from alternative splicing. Examining multiple closely related species also enabled the identification of possible sequencing errors and evolutionary changes. MeaPED enabled the identification of prohormones with potentially miss-specified sequences and the characterization of evolutionary changes associated with neuropeptide specialization and species divergence. Overall, next-generation genome and transcriptome sequencing can be used in non-model species, especially when many similar species are available. These results provide the foundation for experimental identification of peptides associated with social behavior and sex change.

## Supporting information

S1 File Prohormone sequences. Predicted prohormone protein sequences. Predicted protein sequences of the prohormones including isoforms from the different teleost fish species are provided.
(FASTA)

S2 File CARTPT sequences. CART prepropeptide (CARTPT) protein sequences used to generate the phylogenetic tree in Fig 2.
(FASTA)

S3 File PCSK1N sequences. Proprotein convertase subtilisin/kexin type 1 inhibitor (PCSK1N) protein sequences used to generate the multiple sequence alignments in Figs 3 and 4.
(FASTA)

S1 Table Individual prohormone statistics of each prohormone isoform computed for all species, *Pomacentridae* family and *Amphiprion* species. (XLSX)

S2 Table Protein Evolutionary Distance (MeaPED) of each prohormone isoform computed for all species, *Pomacentridae* family and *Amphiprion* species. (XLSX)
